# Identification and Functional Analysis of BmNPV-Interacting Proteins From *Bombyx mori* (Lepidoptera) Larval Midgut Based on Subcellular Protein Levels

**DOI:** 10.3389/fmicb.2020.01481

**Published:** 2020-06-30

**Authors:** Shang-zhi Zhang, Lin-bao Zhu, Dong Yu, Ling-ling You, Jie Wang, Hui-hua Cao, Ying-xue Liu, Yu-ling Wang, Xue Kong, Shahzad Toufeeq, Jia-ping Xu

**Affiliations:** ^1^School of Life Sciences, Anhui Agricultural University, Hefei, China; ^2^Anhui International Joint Research and Developmental Center of Sericulture Resources Utilization, Hefei, China

**Keywords:** *Bombyx mori* (*B. mori*), BmNPV, BmNPV-interacting proteins, subcellular proteins, two-dimensional electrophoresis, far-western blotting

## Abstract

*Bombyx mori* nucleopolyhedrovirus (BmNPV) is a major pathogen causing severe economic loss. However, the molecular mechanism of silkworm resistance to BmNPV and the interactions of this virus with the host during infection remain largely unclear. To explore the virus-binding proteins of silkworms, the midgut subcellular component proteins that may interact with BmNPV were analyzed *in vitro* based on one- and two-dimensional electrophoresis and far-western blotting combined with mass spectrometry (MS). A total of 24 proteins were determined to be specifically bound to budded viruses (BVs) in two subcellular fractions (mitochondria and microsomes). These proteins were involved in viral transportation, energy metabolism, apoptosis and viral propagation, and they responded to BmNPV infection with different expression profiles in different resistant strains. In particular, almost all the identified proteins were downregulated in the A35 strain following BmNPV infection. Interestingly, there were no virus-binding proteins identified in the cytosolic fraction of the silkworm midgut. Two candidate proteins, RACK1 and VDAC2, interacted with BVs, as determined with far-western blotting and reverse far-western blotting. We speculated that the proteins interacting with the virus could either enhance or inhibit the infection of the virus. The data provide comprehensive useful information for further research on the interaction of the host with BmNPV.

## Introduction

The silkworm *Bombyx mori* L. (Lepidoptera: Bombycidae) was domesticated for production of silk fabrics for more than 5000 years. Silkworm is also a good model organism for production of recombinant proteins and the study of insect immunology (Goldsmith et al., 2005; Shao et al., 2012). *B. mori* nucleopolyhedrovirus (BmNPV) is a primary silkworm pathogen, and always causes serious economic losses (Jiang et al., 2012). It is well known that there are two distinct forms of virion in the life cycle of BmNPV: the occlusion-derived virus (ODV) and the budded virion (BV) (Liu et al., 2008). Initially, BmNPV infect silkworm larval midgut cells by ODV, and then infect the larger part of the larva by BVs (Sajjan and Hinchigeri, 2016). During the infection, BmNPV has to rely on host proteins to complete infection and propagation (Kolegraff et al., 2006). Thus, identification of viral binding proteins will have a great significance to clarify the mechanism of silkworm resistance to BmNPV.

Most viruses infect their host by crossing the mucosal surface of the gastrointestinal or respiratory tract. The first step in the virus lifecycle is attachment to a receptor on the host cell membrane, then invasion into cells and spreading to other organs (Sharma et al., 2009). Coxsackieviruses can bind to junction proteins CAR and occludin which are expressed in the tight junction between epithelial cells and infected cells (Coyne et al., 2007). Enveloped virus entry into host cells is typically initiated by an interaction between a viral envelope glycoprotein and a host cell receptor (Zhou and Blissard, 2008). The E3 ubiquitin-protein ligase SINA-like 10 (SINAL10) is a BmNPV-GP64-binding protein that it can stimulates BmNPV proliferation in cells (Feng et al., 2018). Therefore, the search for virus-host interaction proteins is of great significance for the analysis of virus invasion and response of host mechanism.

Until now, many studies have focused on the interaction of virus and host proteins and made a significant progress. It has been reported that four proteins derived from silkworm were identified from BmNPV using two-dimensional electrophoresis (2-DE) combined with mass spectrometry (MS) (Liu et al., 2008). Since then, virus overlay assay also known as Far Western blot has been widely used to identified host proteins that are involved in interacting with virus. Li et al. (2011) reported that five proteins of *Laodelphax striatellus* interacted with rice stripe virus were obtained based on the analysis of 2-DE following by virus overlay assays. Two proteins named SaM35 and SaM50 were identified from the head tissues of the aphid vector as potential receptors for barley yellow dwarf virus using 2-DE following by far-western blot (Li et al., 2001). Importantly, there were 12 proteins of silkworm midgut identified to be specifically bound with BmNPV particles using 2-DE combined with virus overlay assays (Cheng et al., 2014b). However, these researches all focused on the study of the total protein, the resolution is too low to fully identify BmNPV binding proteins.

Recently, the subcellular proteomics has been reported to be a useful strategy to reduce sample complexity and protein overlapping (Lu et al., 2017; Wang et al., 2017). Mitochondria as the “powerhouse of the cell” is involved in many biological processes, including cell growth and death, signal transduction, and cellular differentiation (Schumacker et al., 2014). In addition to their well-appreciated roles in biological processes, mitochondria is associated with many virus infection, such as human immunodeficiency virus (HIV), influenza A virus (IAV), and hepatitis C virus (HCV) (Wu et al., 2013a). Microsomes are vesicle-like artifacts re-formed from pieces of the endoplasmic reticulum (ER), which are involved in ER-like protein synthesis, protein glycosylation, and lipid synthesis. The differentially expressed proteins of microsome are always related to extraneous pathogen infection (Sofra et al., 2009). The cytosol is the liquid found inside cells, where most of the chemical reactions of metabolism take place. Thus, the analysis of the three subcellular proteomics is a very useful strategy to study the interaction of silkworm midgut with BmNPV. However, screening the BmNPV binding proteins from the subcellular protein of silkworm midgut has not been reported.

In this study, one- and two- dimensional electrophoresis following by far-western blot were adopt to identify the viral binding proteins from three subcellular fractions of silkworm midgut: cytosol, mitochondria, and microsome. A total of 24 proteins of silkworm midgut were determined to be specifically bound to BV *in vitro*, which were all identified by MS. Interestingly, there was not any viral binding proteins identified form the cytosolic fraction of silkworm midgut in both the one- and two- dimensional electrophoresis. The relationship of the two candidate proteins of these identified proteins with BmNPV, RACK1, and VDAC2 were further study by using Far-western blot. Overall, this is the first report on identification of viral binding proteins based on the subcellular protein level, which might accelerate our understanding of the pathogenic mechanisms of BmNPV infected silkworm.

## Materials and Methods

### Silkworm and Virus

The susceptible strain P50 (LC50 = 1.03 × 10^5^ OBs/mL) and resistant strain A35 (LC50 = 5.90 × 10^7^ OBs/mL) were maintained in the Key Laboratory of Sericulture, Anhui Agricultural University, Hefei, China (Cheng et al., 2014a). The first three instar larvae were reared on a fresh artificial diet at 26 ± 1°C, 75 ± 5% relative humidity, and a 12 h day/night cycle. The rearing temperature for the last two instars was decreased to 24 ± 1°C, other conditions were as usual. Thirty larval midguts were mixed together to minimize individual genetic differences. Samples were flash-frozen in liquid nitrogen and pulverized. One hundred milligram of each sample was placed directly into RNase-free microcentrifuge tubes containing 1.0 mL of TRIzol Reagent (Invitrogen, United States). The rest samples were kept for protein and DNA extraction. All the samples were stored at −80°C for later use.

The purified BmNPV (T3 strain) was maintained in our laboratory and the concentration was calculated by hemocytometer. Silkworm infected with BmNPV and sample preparation were according to the methods used in our laboratory previously (Wang et al., 2016). The budded virus containing EGFP-tagged (BV-EGFP) BmNPV were kindly provided by Xueyang Wang in School of biotechnology, Jiangsu University of Science and Technology, Zhenjiang, China. The amount of BV-EGFP (pfu/mL) was confirmed by the end point dilution assay method.

### Genome DNA Extraction

The genome DNA of silkworm midgut was extracted according to the method reported by Brevnov et al. (2009) with some modifications. One hundred milligram of sample was dissolved in 1.0 mL lysis buffer (10 mM Tris-HCl pH 8.0, 0.1 M EDTA pH 8.0, 0.5% SDS) with 400 μg/mL final concentration of proteinase K. The sample was incubated at 50°C for 5 h and then mixed with equal volume mixture that was consisted of phenol, chloroform, and isoamylol (25:24:1). After incubating at room temperature (RT) for 30 min, the mixture was centrifuged at 10,000 × g for 10 min at RT. The supernatant was collected and then mixed fully with another equal volume mixture that was consisted of phenol and chloroform (1:1). After incubating at RT for 20 min, the mixture was centrifuged at 10,000 × g for 10 min at RT. The supernatant was collected and then mixed fully with equal volume of chloroform. After incubating at RT for 20 min, the mixture was centrifuged at 10,000 × g for 10 min at RT. The supernatant was transferred into a new microcentrifuge tube and mixed fully with two times volume of precooling absolute ethyl alcohol. The pellet was collected after 10 min centrifugation at 850 × g at RT, and then washed by 70% alcohol for twice. The pellet dried for 6 min at RT was resolved in TE buffer. The product was stored −20°C for later use.

### Extraction of Subcellular Proteins

Subcellular proteins of the P50 strain midgut were extracted according to the method used by Wang et al. (2017). Briefly, the mitochondrial proteins were fractionated in the first centrifugation at 10,000 × g for 10 min at 4°C. Subsequently, the supernatant containing cytosolic and microsomal proteins was mixed with an equal volume of 16 mM CaCl_2_. The microsomal proteins were obtained after centrifuging at 10,000 × g for 10 min. The resulting supernatant containing cytosolic proteins were enriched by 4 × volumes of precooled acetone. Then, the cytosolic proteins were cleaned using the 2-D Clean-up Kit (GE Healthcare, United States) according to the manufacturer’s instructions. The concentration of the three subcellular fractions protein was calculated using the Bradford method (Bradford, 1976).

### Sodium Dodecyl Sulfatepolyacrylamide Gel Electroporesis (SDS-PAGE)

For SDS-PAGE, the protein samples mixed with one fifth volume of 5 × loading buffer containing 50 mM Tris-HCl pH 8.0, 250 mM DTT, 5% SDS, 50% glycerol, and 0.04% bromophenol blue were boiled for 10 min. Subsequently, the samples were separated using SDS-PAGE with a 12% sodium dodecyl sulfate (SDS) polyacrylamide gel. Electrophoresis performed on the Mini-protean Tetra system (Bio-Rad, United States) was proceeded until the bromophenol blue ran out from the bottom of the gels. All the gels were stained with Coomassie brilliant blue R250.

### Two-Dimensional Electrophoresis (2-DE)

For 2-DE, 400 μg of each sample protein dissolved in 125 μL of rehydration solution with 0.3 μL of 1% bromophenol blue dye was loaded onto a 7-cm immobilized linear dry strip (pH 3-10, Bio-Rad, United States). The strip was actively rehydrated at 20°C for 13 h at 50 V. The rehydrated strip was automatically focused using the following program: 200 V, linear, 30 min; 500 V, rapid, 1 h; 4000 V, linear, 3 h; 4000 V, rapid, 20,000 V.h; and 500 V, rapid, 24 h (Cheng et al., 2014b). The current for each strip was limited to 50 μA. After IEF separation, the strips were immediately equilibrated with gentle shaking for 14 min 30 s in equilibration buffer (6 M urea, 20% glycerol, 2% SDS and 0.375 mM Tris-HCl pH 8.8) containing 2% (w/v) DTT, followed by an equilibration for 14 min 30 s in the above equilibration buffer but containing 2.5% (w/v) IAM instead of DTT. Equilibrated IPG strips were further separated on a 10% SDS-PAGE gel. The procedures were performed at 10 mA/gel for 30 min and then 30 mA/gel until the bromophenol blue dye ran out from the bottom of the gels. All the gels were stained with Coomassie brilliant blue R250.

### Virus Overlay Assays (Far-Western Blot)

Followed by electrophoresis, proteins were transferred onto polyvinylidene fluoride (PVDF) membrane (Millipore, United States) using Trans-Blot^®^ SD Cell (Bio-Rad, United States). The virus overlay assays were applied according to the methods reported by Wu et al. (2007) and Cheng et al. (2014b) with some modifications. Briefly, proteins on the membrane were denatured in high concentration the guanidine-HCl and then renatured in gradually reducing the guanidine-HCl concentration. The renatured proteins were subsequently incubated overnight with BmNPV particles. After washed three times in PBST, the membranes were incubated with monoclonal antibodies (MAbs) against *Autographa californica* multicapsid nucleopolyhedrovirus (AcMNPV) gp64 protein (1:500, Santa Cruz). After washing three times, antigen-antibody complexes were detected with goat anti-mouse secondary antibody (1:5000, TransGen biotech, China). After washing three times, the membranes were visualized using a diaminobenzidine (DAB) kit (Tiangen, China) according to the manufacturer’s instructions. For the negative control, the membranes were incubated without BmNPV particles, other steps were all the same. The parallel SDS-PAGE gels and 2-DE gels stained with Coomassie brilliant blue R-250 were used to locate the potential virus-binding proteins.

### MS Analysis and Protein Annotation

After comparing the signals on the membranes with stained gels, the corresponding bands and spots were cut out from the parallel stained SDS-PAGE and 2-DE gels, respectively. The bands and spots were identified using LC-MS/MS and MALDI-TOF/TOF, respectively. The MS analysis was performed by Sangon Biotech (Shanghai, China). Firstly, in-gel digestion was performed as described earlier (Steiner et al., 2000). Briefly, protein gels were de-stained at room temperature by washing with milli-Q water. The de-staining solution was removed and then incubated with different concentrations acetonitrile. Then, the gel was rehydrated in trypsin solution (Promega, Madison, United States). Next, cover solution (25 mM NH_4_HCO_3_) was added for digestion at 37°C for 16 h. The supernatant was extracted once with extraction buffer (67% acetonitrile and 5% TFA). The peptide extract and supernatant were combined and then completely dried. The prepared sample was re-suspended with 0.1% TFA, followed by mixing with a matrix consisting of a saturated solution of α-cyano-4-hydroxy-trans-cinnamic acid in 50% acetonitrile and 0.1% TFA.

For LC-MS/MS, the sample was desalted by loading onto Eksigent nanoLC-Ultra^TM^ 2D system (AB SCIEX, United States) C18 pre nanoflow HPLC column and analyzed by loading onto C18 reverse phase column. LC-MS/MS analysis was carried out using a TripleTOF 5600 system (AB SCIEX, United States) with an iron spray voltage of 2500V. The data was processed under the mode of information dependent analysis (IDA).

For MALDI-TOF/TOF, peptide MS and MS/MS data were obtained with an ABI 5800 MALDI-TOF/TOF plus mass spectrometer (Applied Biosystems, United States). The data was obtained in a positive MS reflector using a CalMix5 standard to adjust the instrument (ABI5800 Calibration Mixture). The GPS Explorer V3.6 software (Applied Biosystems, United States) with default parameters was used to integrate and process both the MS and MS/MS data.

Database searching was performed based on a 95% or higher confidence interval of the scores of proteins in the Mascot V2.3 search engine (Matrix Science Ltd., United Kingdom) using the following parameters: NCBInr database; trypsin as the digestion enzyme; one missed cleavage site; fixed modifications of carbamidomethyl (C); partial modifications of acetyl (Protein N-term), deamidated (NQ), dioxidation (W), oxidation (M); 100 ppm for precursor ion tolerance and 0.5 Da for fragment ion tolerance.

The identified proteins were annotated based on related literatures and information available in various databases, including the NCBI, Swiss-Prot/TrEMBL, and Gene Ontology (GO) databases.

### Protein-Protein Interaction Analysis

The online software STRING^1^ contains abundant resources on physical and functional interactions and collects information from numerous sources, including computational prediction methods, experimental repositories, and public text collections (Jensen et al., 2009). In this study, STRING was adopted to analyze the protein-protein interactions (PPIs) of these BmNPV binding proteins. Due to the lack of proteomics information on *B. mori* in STRING, the PPIs network was mapped using the database of another well-studied insect, *Drosophila melanogaster* (*D. melanogaster*).

### RNA Extraction and cDNA Synthesis

The midgut total RNA was extracted using TRIzol Reagent (Invitrogen, United States) according to the manufacturer’s instructions. The ratios of A260/280 and the concentrations of the total RNA were determined by NanoDrop 2000 spectrophotometer (Thermo Fisher Scientific, United States). The integrity was confirmed by 1% agarose gel electrophoresis. The first strand cDNA was synthesized using PrimeScript RT reagent kit with gDNA Eraser according to the manufacturer’s instructions (TaKaRa, Japan). The internal control primers of *B. mori glyceraldehyde-3-phosphate dehydrogenase* (*BmGAPDH*) were used to evaluate the quality of the cDNA. The qualified cDNA was stored at −20°C for later use.

### Prokaryotic Expression and Purification of Recombinant Protein

The expression of selected genes and purification of fusion proteins were according to the methods reported by Toufeeq et al. (2019) with some modifications. The primers used in the prokaryotic expression are shown in Supplementary Table S1. Briefly, the selected genes were cloned into the pMD-19T vector for sequencing and then into the pET-28a to express recombinant proteins. The recombinant proteins were purified using the high affinity Ni-NTA resin (GenScript, China) according to the manufacturer’s instructions. The purified recombinant proteins were stored at −80°C for later use.

### Reverse Transcription Quantitative PCR (RT-qPCR)

The primers used in the RT-qPCR are shown in Supplementary Table S1. RT-qPCR reactions were prepared with a SYBR Premix Ex TaqTM Kit (TaKaRa, Japan) according to the manufacturer’s instructions. The reactions were carried out in the CFX96TM Real-Time System (Bio-Rad, United States). The thermal cycling profile consisted of an initial denaturation at 95°C for 30 s and 40 cycles at 95°C for 5 s and 60°C for 30 s. All assays were performed in triplicate. Relative expression levels were calculated using the 2^–△△*Ct*^ method (Livak and Schmittgen, 2001). In this study, *BmGAPDH* was selected as an internal control to adjust the data. Statistical analyses were conducted using the SPSS software (IBM, United States).

## Results

### Detection of BmNPV Infection in Different Samples

**FIGURE 1:**
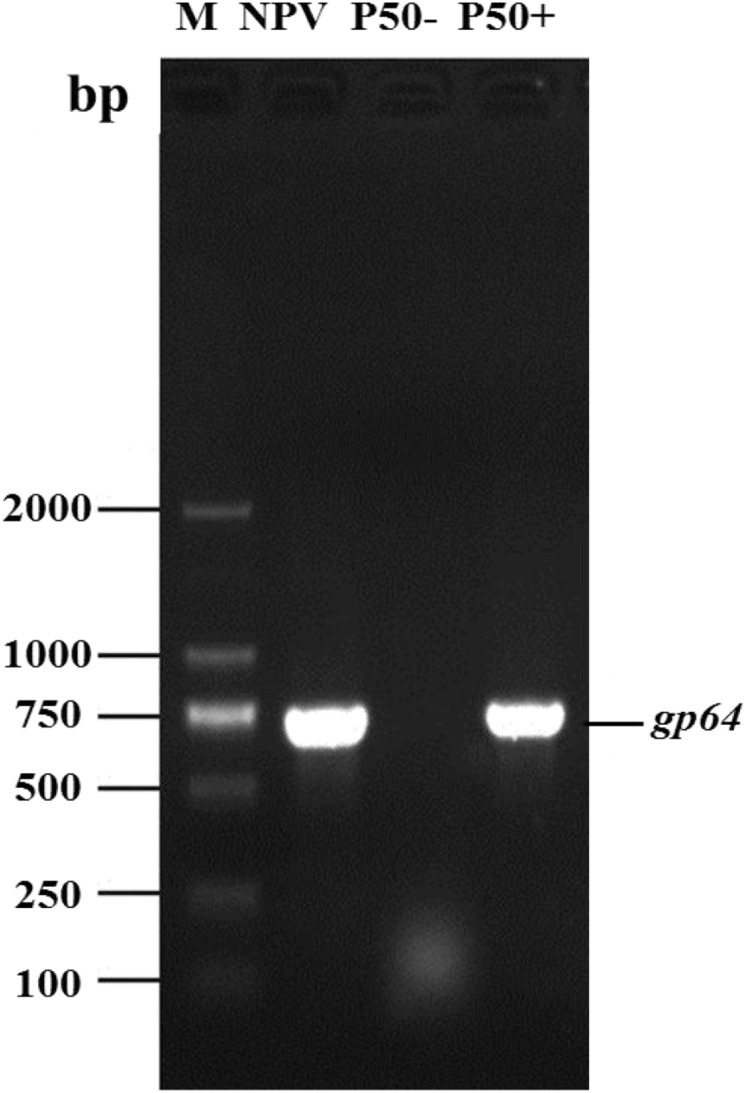
P50 silkworm strains BmNPV free (P50-) and infected (P50+). The DNA of the silkworm midgut was used as a template for baculovirus major protein *gp64* gene PCR amplification. BmNPV DNA was used as a positive control (NPV). M, DNA molecular weight marker.

To determine whether the P50 samples were infected with BmNPV, the expression of the *gp64* of BmNPV in BmNPV-free (P50-) samples and BmNPV infected samples (P50 +) was analyzed using PCR. The primers used for PCR are shown in Supplementary Table S1. Analysis of the PCR products on agarose gels showed that P50 + samples exhibited a clear band, of 750 bp in length, that was not present in the BmNPV-free strains (Figure 1). Thus, the (P50-) samples could be used for the following experiments.

### Variations in Protein Banding Patterns Among the Subcellular Proteins

To improve the resolution of the findings, the total protein of silkworm midgut sample was divided into three subcellular fractions according to methods used by Wang et al. (2017). To check the quality of the subcellular fractions, the subcellular protein fractions were separated by SDS-PAGE. Protein band a in the total protein sample in lane 3 corresponded to the unique mitochondrial band a1 in lane 4. Band b in the total protein lane corresponded to the cytosolic-specific band b1 in lane 6. Band c in the total protein lane corresponded to the microsomal-specific band c1 in lane 5. These results showed that the subcellular proteins were of good quality and suitable for the subsequent analysis (Supplementary Figure S1).

### Interaction Between Mitochondrial Proteins in the Silkworm Midgut and BVs

**FIGURE 2:**
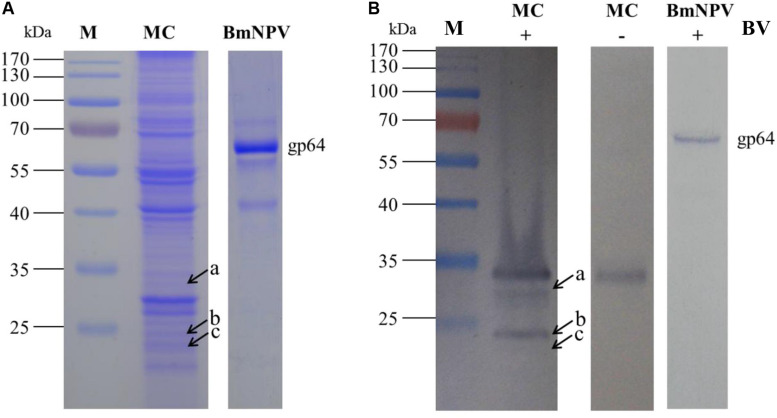
Identification of virus-binding of the mitochondrial proteins fraction of the P50 midgut on SDS-PAGE. **(A)** Separation of *Bombyx mori* midgut mitochondrial (MC) and BmNPV budded viruses (BVs) proteins by SDS-PAGE. **(B)** Virus overlay binding experiment. Mitochondrial protein blots, overlaid with (MC + BV) and without BVs (MC-BV) were incubated with antibodies against the AcMNPV gp64 protein to detect the BVs. BmNPV Budded virus was used as a positive control (BV) to gp64 protein. Arrows a, b, and c in **(A,B)** refer to the bands detected in the stained gel and the PVDF membrane. M, protein molecular weight marker.

To detect the specific binding proteins of silkworm midgut mitochondria to BVs, 1-DE and virus overlay assays were applied. Mitochondrial proteins were separated by 12% SDS-PAGE followed by a BV overlay assay, and three signal bands, named a, b and c, were detected on the PVDF membrane (Figure 2B), as indicated by arrows. To determine the identity of the proteins represented by these three bands, the corresponding bands in the stained gel (Figure 2A) were cut and removed for use in the LC-MS/MS analysis. A total of 12 proteins were identified from the three bands: H + -ATPase β1, H + -ATPase β2, H + -ATPase d, β-tubulin, YWHA, ETF-alpha, SDRs, V-ATPase-A, V-ATPase-B, SP, trypsin, and ATP synthase. Detailed information on these proteins is listed in Table 1.

**TABLE 1:** Identification of BmNPV-binding proteins in *B. mori* midgut mitochondria separated by SDS-PAGE and identified by LC-MS/MS.

Identified proteins	Score	Coverage	NCBI code	Mw (kDa)
**Band a**				
H^+^ transporting atpsynthase beta subunit isoform 2 (H^+^-ATPase β2) [*Bombyx mori*]	1508	44%	gi| 95102940	54.859
Vacuolar ATP synthase subunit B (V-ATPase-B) [*Bombyx mori*]	1329	24%	gi| 118500417	54.667
Beta-tubulin (β-tubulin) [*Bombyx mori*]	945	37%	gi| 3399724	50.582
Tyrosine 3-monooxygenase/tryptophan 5-monooxygenase activation protein epsilon polypeptide protein epsilon polypeptide (YWHA) [*Bombyx mori*]	666	34%	gi| 95102932	29.767
Electron transfer flavoprotein subunit alpha, mitochondrial (ETF-alpha) [*Bombyx mori*]	512	42%	gi| 827563568	34.797
**Band b**				
H^+^ transporting ATP synthase beta subunit isoform 1 (H^+^-ATPase β1) [*Bombyx mori*]	825	31%	gi| 95102938	55.011
Short-chain dehydrogenease/reductase (SDRs) [*Bombyx mori*]	751	29%	gi| 87248225	27.351
Vacuolar ATP synthase catalytic subunit A (V-ATPase-A) [*Bombyx mori*]	588	27%	gi| 119220834	68.558
Serine protease precursor (SP) [*Bombyx mori*]	582	25%	gi| 2116576	30.045
Beta-tubulin (β-tubulin) [*Bombyx mori*]	563	19%	gi| 3399724	50.582
Trypsin, alkaline C-like (Trypsin) [*Bombyx mori*]	516	32%	gi| 512922113	28.430
**Band c**				
H^+^ transporting ATP synthase subunit d (H^+^-ATPase d) [*Bombyx mori*]	668	25%	gi| 95103014	20.190
ATP synthase [*Bombyx mori*]	662	23%	gi| 87248463	59.792
Trypsin, alkaline C-like (Trypsin) [*Bombyx mori*]	532	32%	gi| 512922113	28.430
H^+^ transporting ATP synthase beta subunit isoform 1 (H^+^-ATPase β1) [*Bombyx mori*]	524	28%	gi| 95102938	55.011
Vacuolar ATP synthase subunit B (V-ATPase-B) [*Bombyx mori*]	435	12%	gi| 118500417	54.667

**FIGURE 3:**
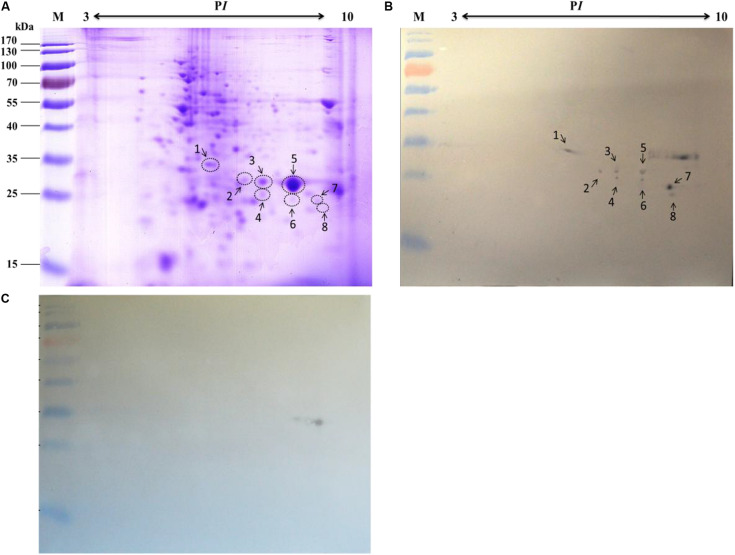
Identification of virus-binding of the mitochondrial proteins fraction of the P50 midgut of the P50 midgut on 2-DE. **(A)** *Bombyx mori* midgut mitochondrial proteins submitted to 2-DE. Mitochondrial proteins blot were overlaid **(B)** or not (**C**; negative control) with BV, and antibodies against the AcMNPV gp64 protein were used to detect the BVs. Arrows 1–8 in **(A,B)** refer to the spots detected on corresponding proteins in the stained gel and the PVDF membrane. M, protein molecular weight marker.

To improve protein resolution, the mitochondrial proteins were separated by 2-DE and subjected to an BV overlay assay. The results showed that a total of 8 spots were detected after overlaid with BV (Figure 3B) and the negative control (Figure 3C) no spot was detected. The corresponding spots in the stained gel (Figure 3A) were cut and removed for use in the MALDI-TOF/TOF MS analysis. Six of these gel samples were successfully identified based on the MS analysis: H + -ATPase β1, VDAC2, SSR-beta, AK2, ECH1, and ATP synthase. Detailed information on the 6 proteins is listed in Table 2.

**TABLE 2:** Identification of BV binding proteins in *B. mori* midgut mitochondria separated by 2-DE and identified by MALDI-TOF/TOF MS.

Spots No.	Identified proteins	Score	Coverage	NCBI code	Mw (kDa)	P*I*
1	H^+^ transporting ATP synthase beta subunit isoform 1 (H^+^-ATPase β1) [*Bombyx mori*]	712	19%	gi| 114052072	54.988	5.26
2	Voltage-dependent anion-selective channel isoform X2 (VDAC2) [*Bombyx mori*]	612	39%	gi| 512928976	30.077	6.96
3	Voltage-dependent anion-selective channel isoform X2 (VDAC2) [*Bombyx mori*]	612	39%	gi| 512928976	30.077	6.96
4	Signal sequence receptor beta subunit precursor (SSR-beta) [*Bombyx mori*]	542	39%	gi| 114052941	20.910	6.91
5	Voltage-dependent anion-selective channel isoform X2 (VDAC2) [*Bombyx mori*]	612	39%	gi| 512928976	30.077	6.96
6	Adenylate kinase 2 (AK2) [*Bombyx mori*]	86	8%	gi| 284813563	26.893	8.85
7	Enoyl-coa hydratase precursor 1 (ECH1) [*Bombyx mori*]	57	6%	gi| 87248109	31.854	8.44
8	ATP synthase [*Bombyx mori*]	316	16%	gi| 114052278	59.658	9.21

### Interaction Between Microsomal Proteins of the Silkworm Midgut and BVs

**FIGURE 4:**
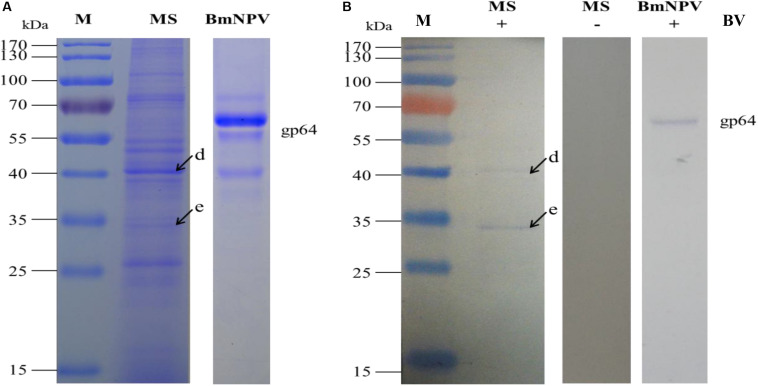
Identification of virus-binding in the microsomal proteins fraction of the P50 midgut on SDS-PAGE. **(A)** Separation of *Bombyx mori* midgut microsomes (MS) and BmNPV budded viruses (BVs) proteins by SDS-PAGE. **(B)** Virus overlay binding experiment. Microsomes protein blots, overlaid with (MS + BV) and without BVs (MS-BV) were incubated with antibodies against the AcMNPV gp64 protein to detect the BVs. BmNPV Budded virus was used as a positive control (BV) to gp64 protein. Arrows d and e in **(A,B)** refer to the bands detected in the stained gel and the PVDF membrane. M, protein molecular weight marker.

The specific binding of the *B. mori* midgut microsomal proteins to BVs was detected as described in the mitochondrial fraction. Microsomal proteins were separated by 12% SDS-PAGE and then a BV overlay assay was applied, and two signal bands, d and e, were detected on the PVDF membrane (Figure 4B), as indicated by arrows. To determine the two protein bands, the corresponding bands in the stained gel (Figure 4A) were cut and removed for use in the LC-MS/MS analysis. Detailed information on the identified proteins from the two bands is listed in Table 3. These proteins were actin4, eEF1 gamma, AST, HIBADH, RACK-1, and LIPH.

**TABLE 3:** Identification of BmNPV-binding proteins in *B. mori* midgut microsomes separated by SDS-PAGE and identified by LC-MS/MS.

Identified proteins	Score	Coverage	NCBI code	Mw (kDa)
**Band d**				
Actin-4 (actin4) [*Bombyx mori*]	2605	44%	gi| 525328733	41.850
Elongation factor 1 gamma (eEF1 gamma) [*Bombyx mori*]	1028	35%	gi| 12328431	48.388
Aspartate aminotransferase (AST) [*Bombyx mori*]	450	36%	gi| 95102552	47.826
**Band e**				
3-hydroxyisobutyrate dehydrogenase isoform X1 (HIBADH) [*Bombyx mori*]	3786	69%	gi| 827560617	34.008
Receptor for activated protein kinase C RACK isoform 1 (RACK-1) [*Bombyx mori*]	989	54%	gi| 87248581	36.041
Lipase member H-A-like (LIPH) [*Bombyx mori*]	680	31%	gi| 512931612	35.925

**FIGURE 5:**
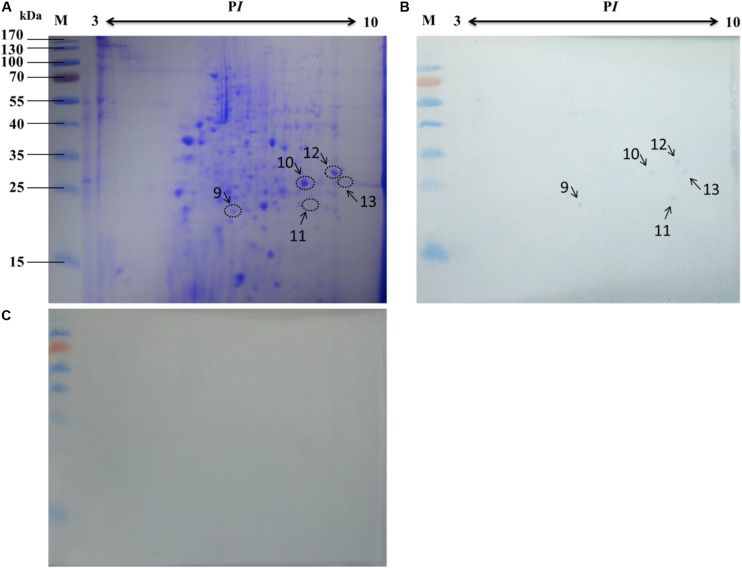
Identification of virus-binding in the microsomal proteins fraction of the P50 midgut on 2-DE. **(A)** *Bombyx mori* midgut microsomal proteins submitted to 2-DE. Microsomal proteins blot were overlaid **(B)** or not (**C**; negative control) with BV, and antibodies against the AcMNPV gp64 protein were used to detect the BVs. Arrows 9–13 in **(A,B)** refer to the spots detected on corresponding proteins in the stained gel and the PVDF membrane. M, protein molecular weight marker.

Based on the 2-DE, a total of 5 spots were detected in the microsomal fraction (Figure 5B), while there was no corresponding spot in the control (Figure 5C). The corresponding spots in the gels (Figure 5A) were cut and removed for use in the MALDI-TOF/TOF MS analysis. All 5 spots were identified based on MS analysis: Acads, ECH1, ECH2, and VDAC2. Detailed information on these proteins is listed in Table 4.

**TABLE 4:** Identification of BmNPV-binding proteins from *B. mori* midgut microsomes separated by 2-DE and identified by MALDI-TOF/TOF MS.

Spots No.	Identified proteins	Score	Coverage	NCBI code	Mw (kDa)	P*I*
9	Short-chain specific acyl-coa dehydrogenase, mitochondrial-like isoform X1 (Acads) [*Bombyx mori*]	484	18%	gi| 512932587	45.143	8.55
10	Enoyl-coa hydratase precursor 1 (mitochondrion) (ECH1) [*Bombyx mori*]	830	36%	gi| 87248111	32.119	8.44
11	Enoyl-coa hydratase precursor 2 (mitochondrion) (ECH2) [*Bombyx mori*]	150	13%	gi| 87248111	30.351	8.46
12	Voltage-dependent anion-selective channel isoform X2 (VDAC2) [*Bombyx mori*]	1038	52%	gi| 512928976	30.115	6.96
13	Enoyl-coa hydratase precursor 1 (mitochondrion) (ECH1) [*Bombyx mori*]	844	36%	gi| 87248111	32.119	8.44

### Interaction Between the Cytosolic Proteins of the Silkworm Midgut and BVs

**FIGURE 6:**
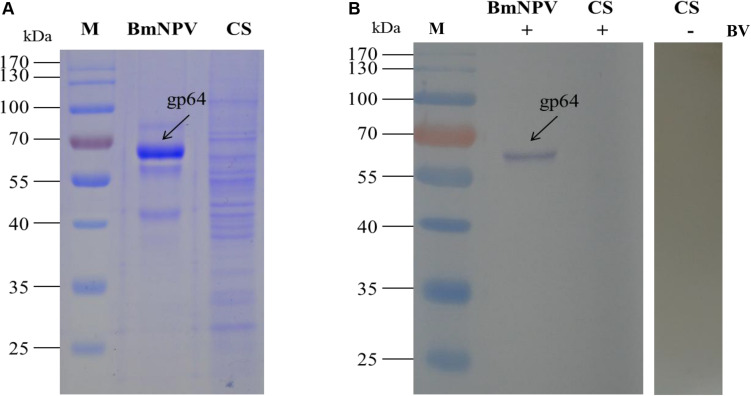
Identification of virus-binding in the cytosol proteins fraction of the P50 midgut on SDS-PAGE. **(A)** Separation of cytosolic proteins of the BmNPV and *Bombyx mori* midgut samples by SDS-PAGE. **(B)** Virus overlay binding experiment. BmNPV and cytosolic protein samples blot were overlaid with BV (CS + BV), and a blot with CS without overlaid with BVs (CS-BV) are detected by antibodies against AcMNPV gp64 protein to detect the BVs. M, protein molecular weight marker.

The specific binding of *B. mori* midgut cytosolic proteins to BVs was detected in mitochondria as described. The cytosolic proteins were separated by 1-DE (Figure 6A) and subjected to an BV overlay assay. However, no corresponding bands were observed on the membrane with the cytosolic samples (Figure 6B). It has been indicated that virus particles cannot not bind with cytosolic proteins in the silkworm midgut. Therefore, we did not run a 2-DE experiment on the cytosolic samples.

### Validation of the BmNPV-Binding Proteins by Far-Western Blotting

**FIGURE 7:**
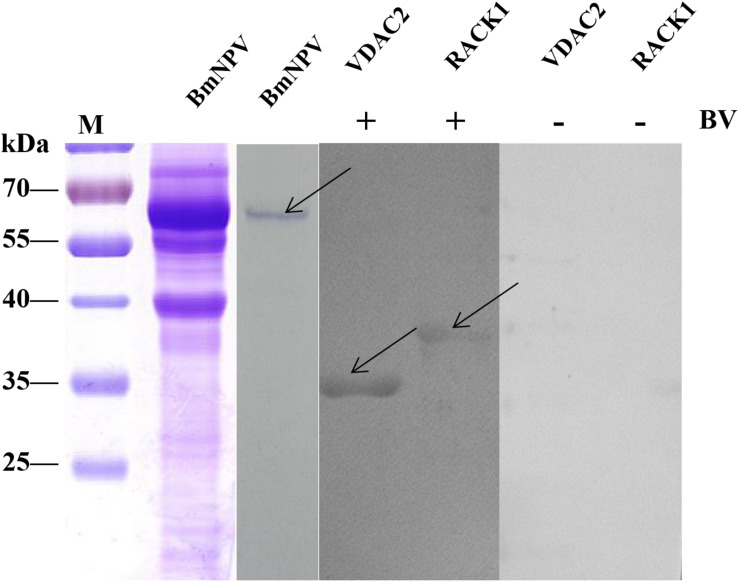
Analysis of the interaction between BVs and recombinant proteins by far western blot. Purified recombinant proteins (RACK1 and VDAC2) were overlaid with BVs, and antibodies against AcMNPV gp64 protein were used to detect the BVs. BmNPV was used as a positive control. The negative overlay control consisted of binding buffer without BVs before incubation with monoclonal antibodies against AcMNPV gp64. The plus and minus signs on the top indicate membranes incubated with or without BVs, respectively. M, protein molecular weight marker.

To validate the BmNPV-binding proteins identified in the silkworm midgut as described above, the candidate proteins RACK1 and VDAC2, which were identified in the mitochondrial and microsomal fractions, were selected for further analysis using far-western blotting experiments. The nucleotide sequences of these two proteins were cloned and ligated into pET-28a to express fusion proteins. The fusion proteins were confirmed with monoclonal antibodies against histidine and purified using high-affinity Ni-NTA resin. To validate the interaction between BmNPV and the two fusion proteins, the purified fusion proteins were separated using 12% SDS-PAGE and then overlaid with BVs. After detection with antibodies against AcMNPV gp64 protein, there were intense signals in the lanes with RACK1 and VDAC2. The results showed that the recombinant proteins RACK1 and VDAC2 could bind to BVs *in vitro* (Figure 7).

**FIGURE 8:**
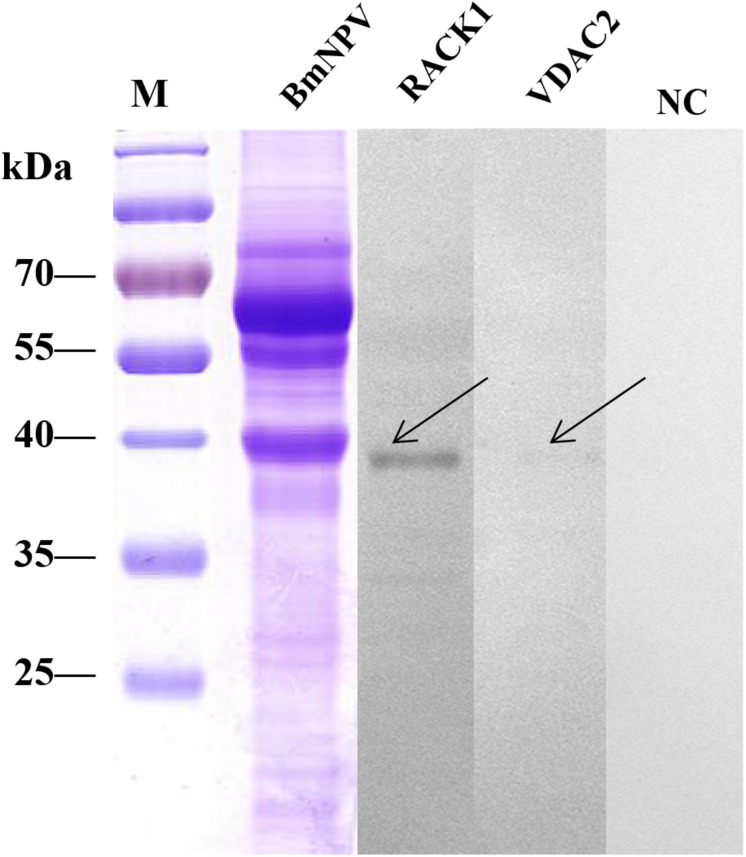
Analysis of the interaction between recombinant proteins and BVs by far western blot. The BVs were overlaid with purified recombinant proteins (RACK1 and VDAC2), and antibodies against His-tagged proteins were used to detect the purified recombinant proteins. M, protein molecular weight marker; NC, negative control, BmNPV incubated with PBS.

Furthermore, the interaction between the proteins of the BmNPV and the two fusion proteins was analyzed using reverse far-western blot experiments. The BmNPV proteins were separated by SDS-PAGE and then transferred to PVDF membranes and incubated with the purified fusion proteins RACK1 and VDAC2. Antibodies against His-tagged proteins were used to detect the fusion proteins. The results showed these proteins were in significant bands in the two groups (Figure 8), which indicated that RACK1 and VDAC2 could bind with BVs *in vitro*.

### Expression Profiles Analysis of Identified Proteins in Different Resistant Strains

**FIGURE 9:**
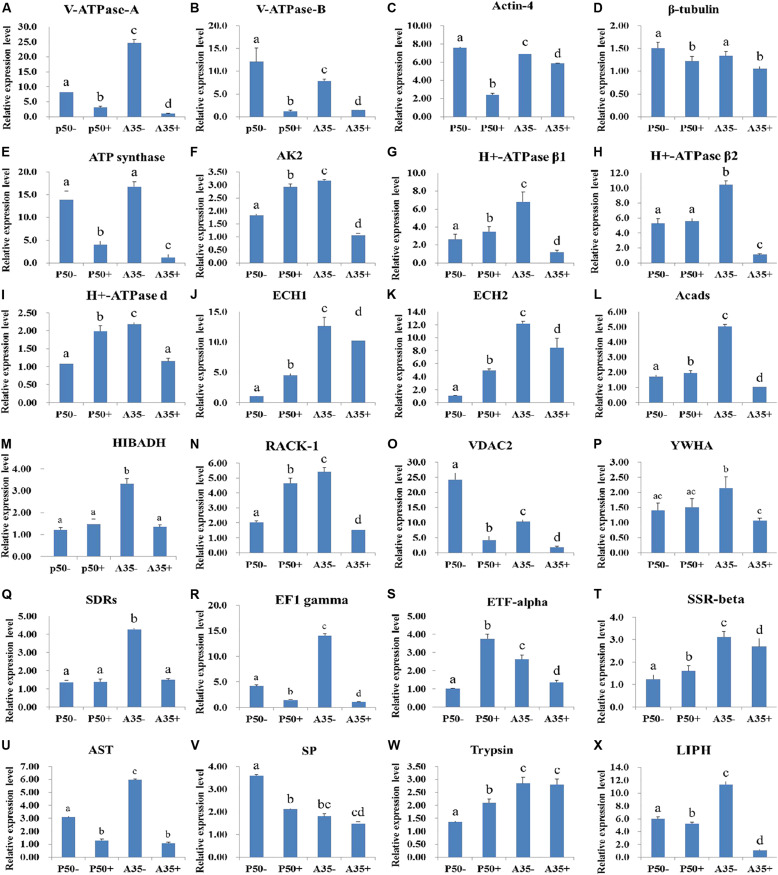
RT-qPCR analysis of the expression profiles of the BmNPV interacting proteins in different resistant silkworm midguts. **(A–X)** represents the transcription level of 24 BmNPVinteracting proteins in different resistant silkworm strains following BmNPV infection. The data were normalized using *BmGAPDH* and presented as the means ± standard error of the means from three independent experiments. The relative expression levels were calculated using the 2^–ΔΔ*Ct*^ method. The statistical analysis was conducted using SPSS software. Significant differences are indicated by letters (*P* < 0.05).

To determine the putative roles of the virus-binding proteins in the BmNPV infection process, the relative expression levels of the 24 corresponding genes in the larval midguts of the susceptible silkworm strain P50 and the resistant silkworm strain A35 following BmNPV infection were analyzed by RT-qPCR. The relative expression levels of 10 genes *V-ATPase-A* (Figure 9A), *V-ATPase-B* (Figure 9B), *actin-4* (Figure 9C), β*-tubulin* (Figure 9D), *ATP synthase* (Figure 9E), *VDAC2* (Figure 9O), *eEF1 gamma* (Figure 9R), *AST* (Figure 9U), *SP* (Figure 9V), and *LIPH* (Figure 9X) were downregulated significantly (*p* < 0.05) in the P50 strain following BmNPV infection at 24 hpi. The other 10 genes, *AK2* (Figure 9F), *H* + *-ATPase*β*1* (Figure 9G), *H* + *-ATPase d* (Figure 9I), *ECH1* (Figure 9J), *ECH2* (Figure 9K), *Acads* (Figure 9L), *RACK-1* (Figure 9N), *ETF-alpha* (Figure 9S), *SSR-beta* (Figure 9T), and *trypsin* (Figure 9W), were upregulated significantly in the P50 strain following BmNPV infection at 24 hpi. The relative expression levels of the four remaining genes, *H* + *-ATPase*β*2* (Figure 9H), *HIBADH* (Figure 9M), *YWHA* (Figure 9P), and *SDRs* (Figure 9Q), were not significantly different in the infected and control P50 strains. It is very interesting that the relative expression levels of all 24 identified genes, except that encoding *trypsin* (Figure 9W), were downregulated significantly (*p* < 0.05) in the A35 + strain midguts compared with the levels in the A35- strain midguts 24 hpi.

## Discussion

Although viruses are simple in structure and composition, their interactions with host cells are complex. The process of virus infection in host cells is based on a repertoire of cellular processes that involve hundreds of cellular proteins (Slack and Arif, 2007). Previous studies reported that many proteins related to the cytoskeleton, cell apoptosis and energy metabolism are all involved in this process, and they may interact with the virus to participate in or respond to this process (Frishknecht and Way, 2001; Harries et al., 2010; Matthews et al., 2013). Although proteomics technology has been widely applied for virus-host interaction studies, to date, little information is available about the protein profile of silkworm larvae infected with BmNPV. Here, subcellular proteomics combined with viral overlay assays was applied to identify BmNPV-binding proteins in the silkworm midgut due to the relatively high resolution offered by this method. Through the analysis of subcellular protein components, not only the number and type of proteins of the research object can be reduced, but also some low-abundance proteins can be effectively enriched. This method can improve the resolution of the analysis and promote the understanding of the expression and function of low-abundance proteins (Lu et al., 2017; Wang et al., 2017). A total of 24 proteins were identified that could bind with BmNPV in the subcellular fractions of silkworm midgut mitochondria and microsomes. We found that these proteins are related to the cytoskeleton, cell apoptosis, energy metabolism and transport in host cells.

Using viral overlay assays combined with LC-MS, we found that 16 and 10 proteins interacted with the virus in mitochondria and microsomes, respectively. However, no proteins in the cytosolic fraction were bound to BVs. Mitochondria are involved in various biological processes, including energy metabolism, cell proliferation and apoptosis (Green and Reed, 1998). The microsome contains the endoplasmic reticulum, which is the main site for protein modification and processing, and serves as the processing station for intracellular proteins (Mandi et al., 2006). A large number of host proteins that are related to multiple biological processes, such as energy metabolism and apoptosis, are involved in the process of virus infection (Wu et al., 1999), which may explain the reason that the virus-binding proteins were identified in the mitochondria and microsome of the silkworm midgut and not in the cytosolic fraction.

Most of these proteins showed different expression profiles in resistant and susceptible silkworm strains following BmNPV infection. In particular, almost all the identified proteins were downregulated in the A35 strain after BmNPV infection. The candidate BmNPV interaction proteins RACK1 and VDAC2 were confirmed. We speculated that, when these proteins interact with the virus, they promote or inhibit the infection of the virus. These data provide comprehensive information to indicate the BmNPV pathogenic mechanisms associated with virulent and silkworm strains.

### Proteins Involved in Virus Transportation

In the process of baculovirus budded viruses (BVs) entering host cells, the acidic environment can regulate the virus-encoded gp64 protein to promote membrane fusion of the virus and endosome and then release the nucleocapsid into the cytoplasm (Blissard and Wenz, 1992; Kingsley et al., 1999; Li et al., 2019b; Xu et al., 2020). V-ATPases are ATP-dependent proton pumps that can maintain the acidification of endosomes (Nishi and Forgac, 2002; Forgac, 2007; Harrison and Muench, 2018). Various enveloped viruses depend on V-ATPase acidification in the endosome for entry (Gruenberg and Van Der Goot, 2006; Marjuki et al., 2011; Li et al., 2019a); one of these is the influenza A virus (IAV) (Guinea and Carrasco, 1995; Ochiai et al., 1995). We speculate that virus interactions with V-ATPases promote virus entry into the cytoplasm. In this study, the expression levels of V-ATPase A and B were significantly downregulated in the A35 and P50 strains following BmNPV infection, which might have been due to the reduction in the expression of V-ATPase A and B, suppressing viral transmembrane transport into the cytoplasm. Once the virus enters the cytoplasm, the viral nucleocapsid depends on the cytoskeleton of the host cell to reach the nucleus and complete replication (Radtke et al., 2006; Volkman, 2007). The cytoskeleton is critical for the maintenance of cell shape, cell motility and intracellular transport, and virus infection requires the cytoskeleton (Harries et al., 2010; Matthews et al., 2013). Two cytoskeletal proteins, actin-4 and β-tubulin, were identified in our study and were both downregulated following BmNPV infection. It has been found that the processes of host infection and replication are related to the cytoskeleton in many viruses, such as *Autographa californica* Multiple nucleopolyhedrovirus and vaccinia virus (Frishknecht and Way, 2001; Zhang et al., 2018). Therefore, these results provide direct evidence that these two cytoskeletal proteins are involved in BmNPV binding and transportation.

### Proteins Involved in Energy Metabolism

The nucleocapsid is unable to undergo independent energy metabolism and depends on the host cellular energy to facilitate the infection (Mercer et al., 2010). It was reported that genes involved in energy metabolism, including fatty acid metabolism and oxidative phosphorylation, were downregulated following haemorrhagic septicemia virus infection, which might indicate a kind of adaptive protection response to regulate energy metabolism for ATP production during viral infections (Xu et al., 2011; Hwang et al., 2016). In this study, several virus-binding proteins involved in energy metabolism were identified: ATP synthase, AK2, H + -ATPase β1, H + -ATPase β2, H + -ATPase d, HIBADH, ECH1, ECH2, and Acads. It was reported that ATP synthase, AK2, and H + -ATPase β1/2/d (Maegawa et al., 2004) are involved in energy metabolism. Recent studies showed that ATP synthase and AK2 mediate the entry of chikungunya virus into host cells (Markaryan et al., 2001; Fongsaran et al., 2014). Additionally, the chemical inhibition of ATP synthase diminished rotavirus (RV) yield in both conventional cell culture and in human intestinal enteroids (Ren et al., 2019). Therefore, these results may indicate that the nucleocapsid needs to bind to these proteins to obtain energy for transport and assembly. Because of the function of these proteins, hosts would benefit from decreased expression of ATP synthase and AK2 to prevent virus infection, which could explain the significant downregulation of the expression levels of five identified proteins (ATP synthase, AK2, and H + -ATPase β1/2/d) in the A35 strain following BmNPV infection. ECH and Acads are involved in catalyzing the β-oxidation pathway of fatty acids and regulating energy homeostasis (Agnihotri and Liu, 2003; Shen et al., 2009). Knocking down the ECH gene apparently impaired wild-type measles virus replication, and ECH was highly associated with genes neighboring Acads (Takahashi et al., 2007; Chen and Su, 2015). Such important roles for ECH1, ECH2, and Acads in virus replication might explain the upregulation of these three proteins in the P50 strain and their downregulation in the A35 strain following BmNPV infection. HIBADH is one of the critical enzymes generating glucose by metabolizing amino acids in the gluconeogenesis pathway, and it is involved in mitochondrial functions (Yao et al., 2010; Tasi et al., 2013). The lower expression level of HIBADH in the A35 strain following BmNPV infection indicates its potential role in inhibiting virus infection.

### Proteins Associated With Apoptosis

Apoptosis is a primitive defense mechanism vital to Lepidopteran insects because they lack humoral immunity, which functions as antiviral defense mechanism. The importance of apoptosis in the cellular defense against virus infections is gaining recognition, because insects resist virus infection by selective apoptosis of the infected cells from the midgut epithelium (Narayanan, 2004; Wu et al., 2013b). In our study, three proteins, RACK1, VDAC2, and YWHA, were determined to interact with BmNPV and are involved in host cell apoptosis. Meanwhile, the interaction between two proteins, RACK1 and VDAC2, and the BmNPV was further confirmed by far-western blot *in vitro*. Similarly, the interaction between RACK1 and *B. mori* cypovirus (BmCPV) was identified by virus overlay assay, and the infectivity of BmCPV was significantly reduced by small interfering RNA-mediated silencing of RACK1 (Zhang et al., 2017). Additionally, inhibition of RACK1 suppressed cell proliferation and induced apoptosis in HCC MHCC97-H cells (Zou et al., 2018). Previous studies have also revealed the involvement of VDAC in the mitochondria-mediated apoptosis pathway (Premkumar and Simantov, 2002; Zheng et al., 2004; Zaid et al., 2005; Li et al., 2016) via its interaction with various apoptosis-related proteins and the regulation of mitochondrial proteins such as cytochrome c (Crompton, 1999; Cheng et al., 2003; Rostovtseva et al., 2005; Abu-Hamad et al., 2006; Halestrap, 2009; Shoshan-Barmatz et al., 2010). In addition, it was reported that RACK1 played an antiapoptotic role during infectious bursal disease virus (IBDV) infection via interaction with VDAC2 and VP5 (IBDV protein) (Lin et al., 2015). Based on their role in apoptosis suppression, the downregulation of these two proteins in the strain A35 following BmNPV infection may promote apoptosis of the infected cells in the midgut epithelium. YWHA, also known as 14-3-3, is involved in the regulation of a number of intracellular processes, including anti-apoptotic pathways (Snow et al., 2008). It was reported that 14-3-3, a phosphoserine-binding molecule, could bind to apoptosis signal-regulating kinase 1 (ASK1), specifically at Ser-967 of ASK1, to inhibit ASK1-induced apoptosis (Zhang et al., 1999; Liu et al., 2001). In our study, the lower expression level of YWHA in the A35 strain following BmNPV infection indicated its role in initiating the activation of ASK1. We speculate that virus infection destroys the normal apoptosis programme of the host and accelerates virus reproduction by binding to proteins related to host apoptosis. Then, the expression level of apoptosis-related genes is downregulated after the virus achieves infection to prevent an increase in apoptosis that would reduce virus invasion in resistant strains.

### Proteins Related to Viral Propagation

A virus is propagated when it enters the host cell and synthesizes a daughter virus with its own DNA and the material of the host cell, and then, the daughter virus is released and enters another host cell (Pham et al., 2012). In this study, five virus-binding proteins were identified in the silkworm midgut: SDRs, eEF1 gamma, ETF-alpha, SSR-beta, and AST. The physiological function of SDRs has been studied widely, and one SDR gene product appears to be involved in the conversion of signaling molecules to either an active or inactive state (Hoffmann and Maser, 2007). In humans, the SDR protein can activate the transcription of human immunodeficiency virus-1 (HIV-1) (Baker et al., 2000). eEF1 gamma is a multidomain protein and is mainly involved in protein biosynthesis. In *Nicotiana benthamiana*, the accumulation of viral coat proteins and the spread of tobacco mosaic virus (TMV) were greatly reduced after eEF1A or eEF1B was silenced (Hwang et al., 2013; Achilonu et al., 2014). Recently, Singaravelu et al. (2010) reported that ETF-alpha is involved in the beta-oxidation of fatty acids and that the subsequent transfer of their electrons mediated hepatitis C virus (HCV) propagation. Based on the function of these three proteins in the viral propagation process, host cells would benefit from a decrease in the expression of these proteins to suppress viral assembly and replication, which may explain the significant downregulation of SDRs, eEF1 gamma, and ETF-alpha in the A35 strain following BmNPV infection. Additionally, SSR-beta is an integral membrane glycoprotein, and overexpression of the wild-type SSR4 allele partially restores glycosylation (Vogel et al., 1990; Losfeld et al., 2014). Nelson et al. (2016) reported that glycosylation of the E1 glycoprotein plays a major role in the pathogenesis of Ross River virus (RRV) by affecting viral virulence and immunopathology in the mammalian host and replication. In the life cycle of viruses, it must interact with host proteins to complete programmed steps. It was reported that AST, one of the host proteins, was essential for bacteriophage GVE2 infection (Chen et al., 2013). Therefore, it is easy to understand the downregulation of SSR-beta and AST in the A35 strain following BmNPV infection. According to the description presented above, we speculate that the host proteins related to virus propagation interact with the virus and can inhibit the proliferation of the virus in the A35 strain.

### Protease Relevant Proteins

In recent years, many silkworm proteases have been reported to show significant antiviral activity, such as arginine kinase and serine protease (SP-2) (Kang et al., 2011; Qin et al., 2012). In this study, SP was also identified in the silkworm midgut based on a virus overlay assay, and its expression level was downregulated in the P50 strain following BmNPV infection but did not show significantly altered in the infected A35 strain. In addition, alkaline trypsin, a kind of protease, is in the SP family. Ponnuvel et al. (2012) reported that alkaline trypsin purified from the digestive juice of silkworm larvae had strong antiviral activity against BmNPV under *in vitro* conditions. Therefore, the notably higher expression level of trypsin and alkaline C-like (trypsin) in the midgut of the BmNPV-resistant A35 strain than that in the susceptible P50 strain indicates its important role in resisting infection. Zhao et al. (2010) found that SPs were up- or downregulated in silkworms following BmNPV infection, indicating that these SPs play different roles in BmNPV infection. In our study, lipase member H-A-like (LIPH) was also identified in the silkworm midgut. Bmlipase-1 from the digestive juice of silkworms was confirmed to have strong antiviral activity against BmNPV under *in vitro* conditions (Ponnuvel et al., 2003). Interestingly, the expression level of LIPH in the A35 strain was higher than that in the P50 strain, indicating its role in silkworm resistance to BmNPV infection. Previous research showed that proteases potentially act directly on BmNPV by destroying viral integrity and consequently reducing the infectivity of BmNPV (Ponnuvel et al., 2003; Liu et al., 2018). Based on the above results, we speculate that these proteases may influence BmNPV infection by interacting with BmNPV.

### Protein-Protein Interaction (PPI) Network Analysis of the Virus-Binding Proteins

**FIGURE 10:**
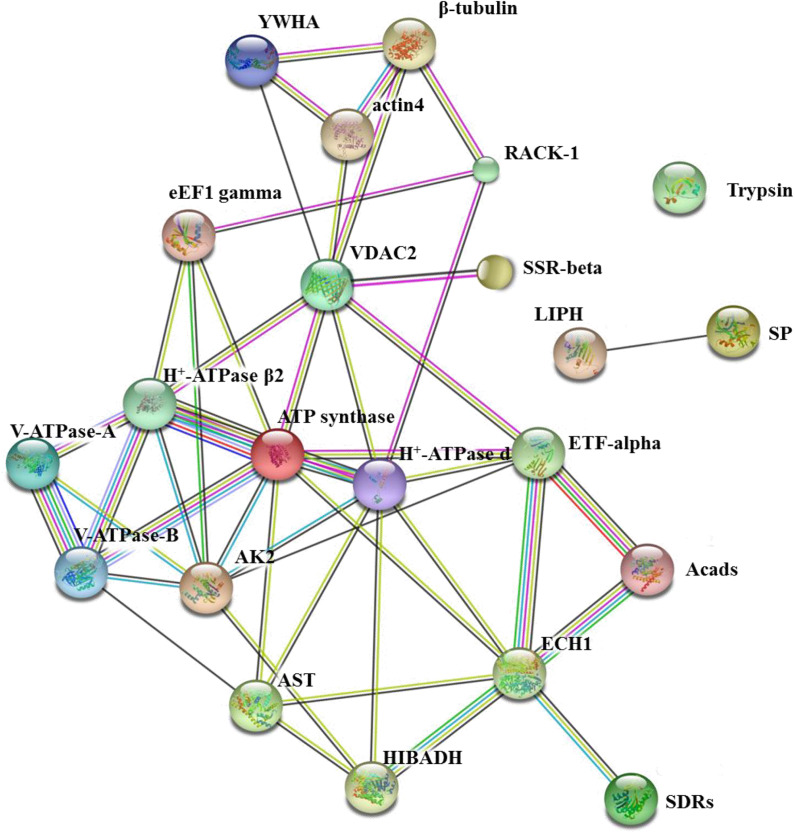
The network of 24 BmNPV-interacting proteins was mapped based on the STRING website information using a database of information on another well-studied insect, *D. melanogaster*. VDAC2, voltage-dependent anion-selective channel isoform X2; SSR-beta, signal sequence receptor beta subunit precursor; AK2, adenylate kinase 2; actin4, actin-4; eEF1 gamma, elongation factor 1 gamma; AST, aspartate aminotransferase; HIBADH, 3-hydroxyisobutyrate dehydrogenase isoform X1; RACK1, receptor for activated protein kinase C RACK isoform 1; LIPH, lipase member H-A-like; Acads, short-chain specific acyl-CoA dehydrogenase; ECH1, enoyl-coa hydratase precursor 1; H + -ATPase β2, H + transporting ATP synthase beta subunit isoform 2; V-ATPase-B, vacuolar ATP synthase subunit B; β-tubulin, beta-tubulin; YWHA, tyrosine 3-monooxygenase/tryptophan 5-monooxygenase activation protein epsilon polypeptide; ETF-alpha, electron transfer flavoprotein subunit alpha; SDRs, short-chain dehydrogenase/reductase; V-ATPase-A, vacuolar ATP synthase catalytic subunit A; SP, serine protease precursor; Trypsin, trypsin, alkaline C-like; H+-ATPase d, H+transporting ATP synthase subunit.

In living cells, many proteins can interact with each other, and these interacting proteins are expected to be involved in the same biological process, which is supported by the evidence that proteins in the same pathway are more interconnected (Barabasi et al., 2011). To investigate the relationship among these BmNPV binding proteins, their functional association was analyzed using STRING 9.1 online software. According to the software, the score of each protein-protein association is calculated by combining the probabilities from several pieces of evidence and correcting for the probability of randomly observing a particular interaction. Based on the analysis of STRING software, most of the BmNPV-binding proteins we identified, except for trypsin, LIPH, and SP, could be mapped into one network with medium confidence (Figure 10). We speculate that these three proteases may be secreted into the lumen from midgut cells, where they can interact with the virus that has entered the lumen to perform their functions. In the BV transmembrane process, V-ATPase-A and V-ATPase-B can interact with each other to promote nucleocapsid release into the cytoplasm. Subsequently, the nucleocapsid enters the nucleus easily facilitated by actin4 and β-tubulin. eEF1-gamma, SDRs, SSR-beta, and ETF-alpha are involved in viral propagation, but their functions in viral propagation are different from each other; for example, viruses depend on eEF1-gamma for replication, and SDRs promote viral transcription. Apoptosis-related proteins YWHA and RACK1 depend on the cytoskeleton and energy to perform their functions. Although these proteins do not connect with each other, they have a close relationship with energy metabolism-related proteins, including ATP synthase, H + -ATPase β2, H + -ATPase d, ECH1, Acads, VDAC, and HIBADH, which play a central role in connecting with these BmNPV-interacting proteins. It is reasonable that all life activities depend on energy, even during the process of virus infection.

Taken together, the finding of this study indicates the virus-binding proteins based on the subcellular proteomics of silkworm midguts. A total of 24 proteins of the silkworm midgut were determined to be specifically bound to BVs from two subcellular fractions (mitochondrial and microsomal) by far-western blotting *in vitro*. These proteins were involved in viral transportation, energy metabolism, apoptosis and viral propagation, and most of these proteins respond to BmNPV infection with different expression profiles in different resistant strains. Interestingly, no virus-binding proteins were identified in the cytosolic fraction of the silkworm midgut. Although the functions of these proteins were not fully verified individually, these substantial observations lays the foundation for in-depth studies that can elucidate the molecular mechanism of the interactions between the silkworm midgut proteins and BmNPV.

## Data Availability Statement

The datasets presented in this study can be found in online repositories. The names of the repository/repositories and accession number(s) can be found in the article/Supplementary Material.

## Author Contributions

SZ and JX conceptualized the study. SZ, LZ, and DY contributed to data curation. SZ, LZ, DY, YL, and JW carried out the investigation. SZ, YW, and DY worked on the methodology. SZ, JW, YL, ST, XK, and LY helped with the resources. LZ, HC, and LY were responsible for the software. JX supervised the study. SZ and JX reviewed and edited the manuscript. All authors contributed to the article and approved the submitted version.

## Conflict of Interest

The authors declare that the research was conducted in the absence of any commercial or financial relationships that could be construed as a potential conflict of interest.

## Footnotes

1 http://string-db.org/
