# Supplementary material for: Identification and Functional Analysis of BmNPV-Interacting Proteins From Bombyx mori (Lepidoptera) Larval Midgut Based on Subcellular Protein Levels
Source: Front Microbiol. 2020 Jun 30;11:1481. doi: 10.3389/fmicb.2020.01481 (PMC7338592; doi:10.3389/fmicb.2020.01481)
Supplement: Supplementary file 1 [file Data_Sheet_1.PDF]

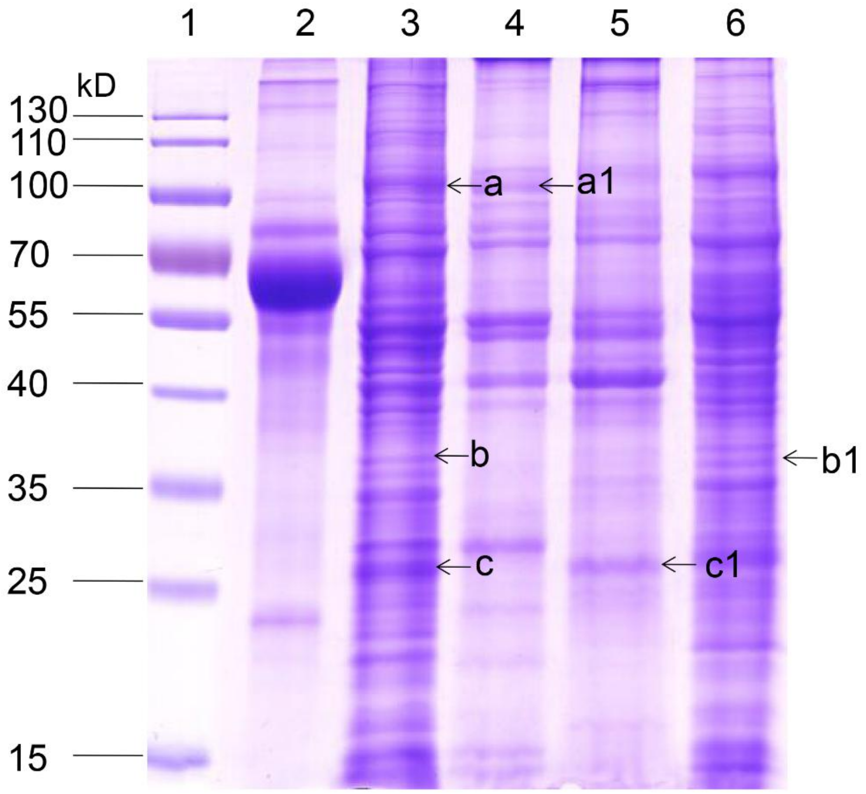

Supplementary Figure 1. Analysis of the subcellular protein fractions of the P50 midgut sample by SDS-PAGE: 1, markers; 2, BV proteins; 3, total proteins; 4, mitochondrial proteins; 5, microsomal proteins; and 6, cytosolic proteins.A quantity of 35 μg of each protein sample was loaded and electrophoresed in a 12% polyacrylamide gel and stained with Coomassie brilliant blue R250. Arrows represent different and corresponding bands in the subcellular fractions.
