# Supplementary material for: Identification and Functional Analysis of BmNPV-Interacting Proteins From Bombyx mori (Lepidoptera) Larval Midgut Based on Subcellular Protein Levels
Source: Front Microbiol. 2020 Jun 30;11:1481. doi: 10.3389/fmicb.2020.01481 (PMC7338592; doi:10.3389/fmicb.2020.01481)
Supplement: Supplementary file 2 [file Table_1.DOC]

**Table S1.** Primers used in this study

| **Primer names** | **Sequence (5'-3')** | **Restriction enzyme** | **Purpose** |
| --- | --- | --- | --- |
| *BmGAPDH* | F: CCGCGTCCCTGTTGCTAAT  R: CTGCCTCCTTGACCTTTTGC |  | RT-qPCR |
| *H+ transporting ATP synthase beta subunit isoform 1* | F: GGACGCATCATCAATGTAATCG  R: AAACAACCCAATCTTTCCACCT |  | RT-qPCR |
| *Voltage-dependent anion-selective channel isoform X2* | F: ATTGTCTTGTGGTGGGCAGT  R: CGCTCTTGGTCTTCAGGTCG |  | RT-qPCR |
| *Signal sequence receptor beta subunit precursor* | F: GCTGTTGAAGTGAAACTCGTGG  R: TTCGCTGGCTTTGTATGTGAC |  | RT-qPCR |
| *Adenylate kinase 2* | F: GCAGCCACGAAACTAAAACCT  R: GCCCGATGAGACTTCAGCAC |  | RT-qPCR |
| *Enoyl-coa hydratase precursor 1* | F: GCAACAAGCCAAGCATCCA  R: GCTGTCGGCGTCAAAATCAT |  | RT-qPCR |
| *ATP synthase* | F: CAGTTCGGTTCTGACTTGGAT  R: AAGGCAGTGATTTTGGAGGG |  | RT-qPCR |
| *Actin-4* | F: GCGTGATGGTCGGTATGGG  R: GCGGGCGTGTTGAATGTTT |  | RT-qPCR |
| *Elongation factor 1 gamma* | F: TCCCGCTGTCGTCTGATTG  R: TGGTTGAACTTTCTACCGTCTTTG |  | RT-qPCR |
| *Aspartate aminotransferase* | F: GCTCGGACATTCCTCGTCTTTT  R: GCGGACCCATCTGAACATTG |  | RT-qPCR |
| *3-hydroxyisobutyrate dehydrogenase isoform X1* | F: CAATAGATCCGAATGTTCC  R: ATGACTCCTCCAGATACA |  | RT-qPCR |
| *Receptor for activated protein kinase C RACK isoform 1* | F: GCAACTAATCCGAAATACCCG  R: GAAATGAAGTGCGAATGACCG |  | RT-qPCR |
| *Lipase member H-A-like* | F: GTGATGCCGACTTCTACCCG  R: ACCACAGCGTCTTCCAACCA |  | RT-qPCR |
| *Short-chain specific acyl-coa dehydrogenase* | F: AATGAGTGTCAGCGCTTGTTCTA  R: AGCCGTCTCCACCAGTATGC |  | RT-qPCR |
| *Enoyl-coa hydratase precursor 1* | F: GCAACAAGCCAAGCATCCA  R: ACGCCTTCTCGTTACCAGTGA |  | RT-qPCR |
| *Enoyl-coa hydratase precursor 2* | F: GCAACAAGCCAAGCATCCA  R: GCTGTCGGCGTCAAAATCAT |  | RT-qPCR |
| *H+ transporting atpsynthase beta subunit isoform 2* | F: ACCTCCTGGTGCCCGTG  R: ATAAATAGCCTGTACAGATGTGATGG |  | RT-qPCR |
| *Vacuolar ATP synthase subunit B* | F: GCCGTGGTAGGTGAGGAGG  R: TGGGGAAGATACGCAGCAAC |  | RT-qPCR |
| *Beta-tubulin* | F: TTCCCCCTCGTGGTCTCAA  R: CCTCGTCAAACTCTGCGTCC |  | RT-qPCR |
| *Tyrosine 3-monooxygenase/tryptophan 5-monooxygenase activation protein epsilon polypeptide* | F: ACAACCTCACGCTGTGGACC  R: ACTGCGGGCTGGCTCTTA |  | RT-qPCR |
| *Electron transfer flavoprotein subunit alpha* | F: GCCGAGCACAACAATGAGG  R: CCAGCCACAAGCACTGAGACA |  | RT-qPCR |
| *H+ transporting ATP synthase beta subunit isoform 1* | F: GTGTCTGCTCTGCTTGGTCG  R: CCTTTCTTGGTGGTGGTAAT |  | RT-qPCR |
| *Short-chain dehydrogenease/reductase* | F: AAGGGGAGGGTTGAGTGAGG  R: CCAAGTCGCTGGCAAGGAA |  | RT-qPCR |
| *Vacuolar ATP synthase catalytic subunit A* | F: AGTTCAAAATGGCGAGCAAAG  R: CGACGGGTCCAGATACGG |  | RT-qPCR |
| *Serine protease precursor* | F: GTCAGTTCACCCTCGCTCTTG  R: GGCTCACTTGGCGTTTTTGTT |  | RT-qPCR |
| *Trypsin, alkaline C-like* | F: GGCTGTTCCTACCAACCCAC  R: CACCACAAGACTGCCACCACT |  | RT-qPCR |
| *H+ transporting ATP synthase subunit d* | F: ATCCACCTGAACCGCCCA  R: GCATTCTTGACTTGATTCCACTG |  | RT-qPCR |
| *Receptor for activated protein kinase C RACK isoform 1* | F: CGCGGATCCGAGTCGTCGCTCTTCAAACC | *BamH I* | Amplification of RACK1 for Prokaryotic expression |
|  | R: CCGGAATTCAGCTTATCGAGCTGAGATGGA | *EcoR I* |  |
| *Voltage-dependent anion-selective channel isoform X2* | F: CGCGGATCCGACATGGCTCCCCCATATTA | *BamH I* | Amplification of VDAC2 for Prokaryotic expression |
|  | R: CCCAAGCTTTTTCTAGGGCTCGAGTTCGA | *Hind III* |  |
| *GP64* | F: CAATCACTTTGCGTACCACA |  | PCR |
|  | R: GCCCTTCTTTGTAAATGCTG |  |  |
